# Illumina sequencing of the chloroplast genome of common ragweed (*Ambrosia artemisiifolia* L.)

**DOI:** 10.1016/j.dib.2017.10.009

**Published:** 2017-10-07

**Authors:** Erzsébet Nagy, Géza Hegedűs, János Taller, Barbara Kutasy, Eszter Virág

**Affiliations:** a University of Pannonia, Georgikon Faculty, Department of Plant Science and Biotechnology, Keszthely, Hungary; b University of Pannonia, Georgikon Faculty, Department of Economic Methodology, Keszthely, Hungary

**Keywords:** Illumina sequencing, Chloroplast genome, cpDNA, Common ragweed, *Ambrosia artemisiifolia*

## Abstract

Common ragweed *(Ambrosia artemisiifolia* L.*)* is the most widespread weed and the most dangerous pollen allergenic plant in large areas of the temperate zone. Since herbicides like PSI and PSII inhibitors have their target genes in the chloroplast genome, understanding the chloroplast genome may indirectly support the exploration of herbicide resistance and development of novel control methods. The aim of the present study was to sequence and reconstruct for the chloroplast genome of *A. artemisiifolia* and establish a molecular dataset. We used an Illumina MiSeq protocol to sequence the chloroplast genome of isolated intact organelles of ragweed plants grown in our experimental garden. The assembled chloroplast genome was found to be 152,215 bp (GC: 37.6%) in a quadripartite structure, where 80 protein coding genes, 30 tRNA and 4 rRNA genes were annotated in total. We also report the complete sequence of 114 genes encoded in *A. artemisiifolia* chloroplast genome supported by both MIRA and Velvet *de novo* assemblers and ordered to *Helianthus annuus* L. using the Geneious software.

**Specifications Table**

**Table:** 

Subject area	*Biology*
More specific subject area	*Chloroplast genome of common ragweed*
Type of data	*Table, figure*
How data was acquired	*2* × *300 Illumina MiSeq sequencing*
Data format	*Raw reads in FASTAQ, complete cp genome in FASTA*
Experimental factors	*5 g young leaves were collected from young about 20 cm tall plants, and incubated for 48* *h at 4 °C in dark*
Experimental features	*Complete chloroplast genome of Ambrosia artemisiifolia*
Data source location	*Keszthely-city, Hungary*
Data accessibility	*Information and complete data are accessible in the NCBI under BioProject and BioSample ID: PRJNA383307, SAMN06761249. The raw reads are available in Fastq format in the NCBI SRA database at the following link*https://trace.ncbi.nlm.nih.gov/Traces/sra/sra.cgi?run=SRR6050242.
	*Complete chloroplast genome is available in GenBank under accession number:*MF362689; https://www.ncbi.nlm.nih.gov/nuccore/MF362689

**Value of the data**

• Common ragweed is one of the most aggressive invasive weed species and the most dangerous pollen allergenic plant in large areas of the temperate zone.
• Understanding the chloroplast genome of this species may indirectly support chemical control of it, since a large part of herbicides have their target genes in the chloroplast genome e.g. triazine-derivatives [1], diphenylethers [2] or the redox active Paraquat [3].
• The reported data mean an important source for further chloroplast derived investigations like phylogenetic, photosynthetic or oxidative metabolism studies of the species.

## 1 Data

Intact chloroplasts were isolated from young leaves of *Ambrosia artemisiifolia*. Followed by cpDNA isolation and sequencing. The raw reads are available in Fastq format in the SRA database under the accession SRR6050242. The assembled chloroplast genome and annotated genes are available through NCBI nucleotide (MF362689).

## 2 Experimental design, materials and methods

### 2.1 Plant material and isolation of cpDNA

Seeds of an *A. artemisiifolia* plant grown in our experimental garden were sown on peat, and plants were grown in pots under greenhouse conditions.

**Fig. 1:**
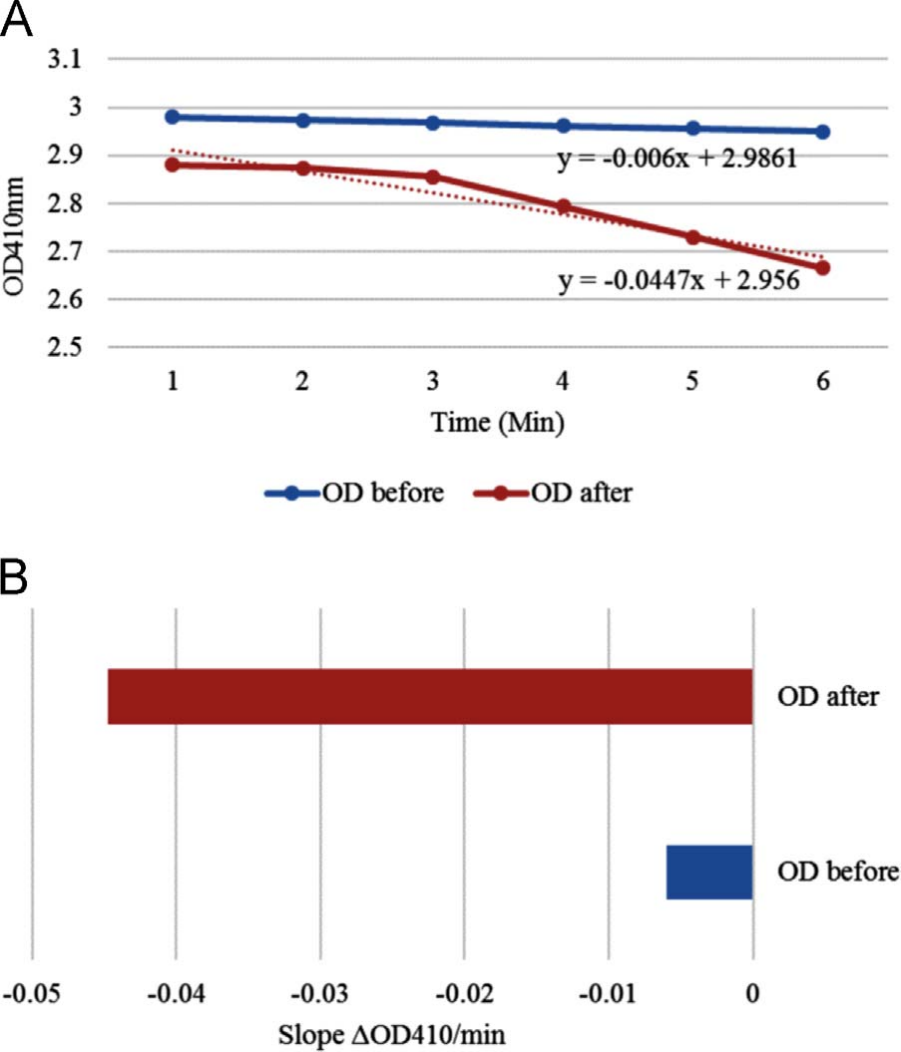
Analysis of the *Ambrosia* chloroplast preparation. Graph A: The degree of integrity of prepared chloroplast. It is assessed by comparing the rate of ferricyanid reduction upon illumination (at 410 nm) before (blue) and after (orange) osmotic shock. Graph B: Bars representing the slopes of the lines in graph A. The differences of slope values indicated that 81% of isolated chloroplast was intact and suitable for cpDNA extraction.

In total, 5 g leaf tissue was collected from young, about 20 cm tall plants. To avoid high level starch accumulation the harvested leaves were incubated in Parafilm-sealed Petri dishes for 48 h at 4 °C in dark before chloroplast preparation. Chloroplast was isolated using the Chloroplast Isolation kit (Sigma-Aldrich, USA) according to the instructions of the manufacturer. The intact chloroplasts were separated from the broken ones by centrifugation on top of 40/80% Percoll® gradient. To calculate the percentage of intact chloroplasts the ferricyanide photoreduction procedure was used [4]. The reduction of ferricyanid was measured spectrophotometrically at 410 nm. The percentage of intact chloroplasts of the preparation was assessed by comparing the rates of ferricyanide photoreduction with and without osmotic shock of the chloroplasts using the following formula:

$$\frac{B-A}{B}\times 100\%=\%\;\mathrm{intact}\;\mathrm{chloroplasts}$$

where *A* and *B* are the change in absorbance at 410 nm as a function of time (min) without and with osmotic shock measured by spectrophotometer. Analysis indicated that the 81% of the Ambrosia chloroplast preparation was intact and suitable for cpDNA extraction (Fig. 1).

Isolation of cpDNA was performed as described by Nascimento Vieira et al. [5] with the following modification: after the addition of potassium acetate the sample was kept on ice for 2 hours. Then the procedure was continued according to reference till DNA dissolution in nuclease-free water.

### 2.2 Library preparation and sequencing

An Illumina paired-end cpDNA library (average insert size of 500 bp) was constructed using the Illumina TruSeq library preparation kit according to manufacturer's protocol. The cpDNA library was sequenced with 2 × 300 bp on MiSeq platform (Illumina, USA).

### 2.3 Chloroplast genome assembly

Prior to the de novo assembly of cp genome quality control of the raw paired-end reads (972,060 reads) were done using FastQC [6]. Based on FastQC report the trimming of low quality sequences (quality score < 20; Q20) were filtered out by using a self-developed application, GenoUtils, written in Visual Studio integrated developmental environment with C#. The remaining high quality paired end reads (864,583 reads) were assembled. To create full-length contiguous sequences without the guidance of a reference genome, we obtained *de novo* assembly by applying the overlap-based genome assembler MIRA (version 4.0.2) [7] and Velvet (version 1.2.10) [8]. The assembled contigs were ordered against the complete cp genome of *Helianthus annuus* L. as reference using the Geneious (version 9.1.6) (http://www.geneious.com) software [9].

### 2.4 Gene annotation

The web-based program Dual OrganellarGenoMe Annotator (DOGMA, http://dogma.ccbb.utexas.edu/) [10] was used to annotate the assembled genome using default parameters to predict protein coding genes, as well as tRNA and rRNA genes. The previously reported *A. artemisiifolia* transcriptome dataset [11] was used to identify the coding regions of cp genes [2]. Subsequently, BLASTN was used to further identify intron-containing gene positions by searching the *de novo* assembled cp genome.

**Fig. 2:**
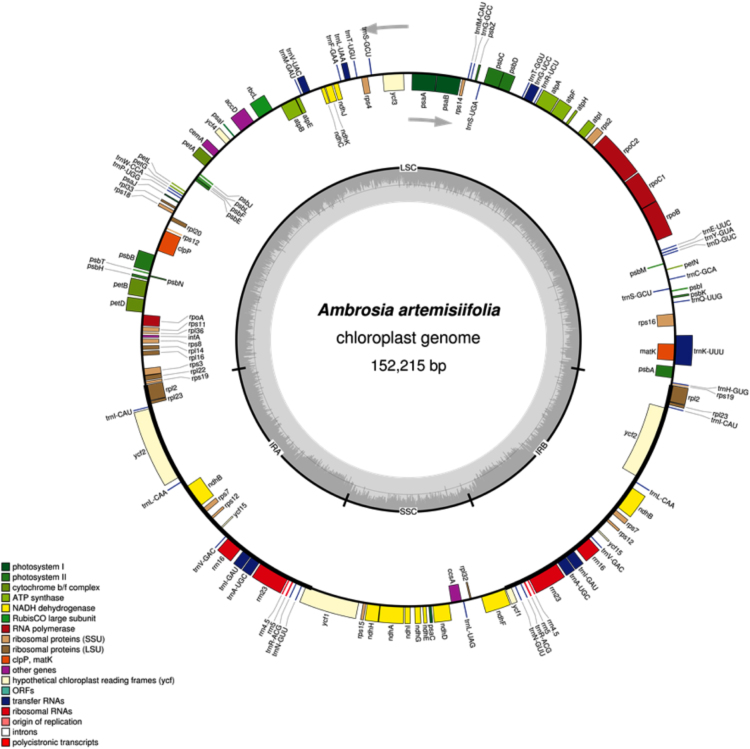
Physical map of *Ambrosia artemisiifolia* cp genome. The graphical organization was created by **OGDRAW**[12].

The size of the complete chloroplast genome of *A. artemisiifolia* was found to be 152,215 bp (GC: 37.6%). The cp genome exhibited a quadripartite structure consisting of LSC and SSC regions of 84,399 bp and 17,958 bp respectively, separated by a pair of inverted repeats (IRa and IRb) each being 24,929 bp. A total of 114 genes were annotated including 80 protein coding genes, 30 tRNA genes, and 4 rRNA genes. Six of the protein coding genes and the 3' exon of rps12 are duplicated in the IR regions. Seven of the tRNA genes and all four rRNA genes are also duplicated in the IR regions. The presence of one or two introns were identified in 16 genes, which include 10 protein coding genes and six tRNA genes (Table 1, Fig. 2).

**Table 1:** Classification of genes after chloroplast genome reconstruction. The annotated genes were categorized according to their function. Nominations: underlined: contains one intron, underlined bold: **contains more than one intron**.

**Group of genes**	**Genes**
***Protein genes***	
ATP synthase	*atpA atpB atpE atpF atpH atpI*
Cytochrome b/f complex	*petA petB petD petG petL petN*
Large subunit of RuBisCO	*rbcL*
NADH dehydrogenase	*ndhA ndhB ndhC ndhD ndhE ndhF ndhG ndhH ndhI ndhJ ndhK*
Photosystem I.	*psaA psaB psaC psaI psaJ*
Photosystem II.	*psbA psbB psbC psbD psbE psbF psbH psbI psbJ psbK psbL psbM psbN psbT psbZ*
Photosystem I assembly protein	***ycf3** ycf4*
Proteins of unknown function	*ycf1 ycf2 ycf15*
Ribosomal proteins	
Large subunit	*rpl2 rpl14 rpl16 rpl20 rpl22 rpl23 rpl32 rpl33 rpl36*
Small subunit	*rps2 rps3 rps4 rps7 rps8 rps11 rps12 rps14 rps15 rps16 rps18 rps19*
RNA polymerase	*rpoA rpoB rpoC1 rpoC2*
Translation factor	*infA*
Other genes	*accD cemA **clpP** ccs matK*
**RNA genes**	
Ribosomal RNAs	*rrn4.5 rrn5 rrn16 rrn23*
Transfer RNAs	*trnA-UGC trnC-GCA trnD-GUC trnE-UUC trnF-GAA trnfM-CAU trnG-GCC trnG-UCC trnH-GUG trnI-CAU trnI-GAU trnK-UUU trnL-CAA trnL-UAA trnL-UAG trnM-CAU trnN-GUU trnP-UGG trnQ-UUG trnR-ACG trnR-UCU trnS-GCU trnS-GCU trnS-UGA trnT-GGU trnT-UGU trnV-GAC trnV-UAC trnW-CCA trnY-GUA*
