# Supplementary material for: Illumina sequencing of the chloroplast genome of common ragweed (Ambrosia artemisiifolia L.)
Source: Data Brief. 2017 Oct 7;15:606–11. doi: 10.1016/j.dib.2017.10.009 (PMC5655400; doi:10.1016/j.dib.2017.10.009)
Supplement: Supplementary file 1 — Transparency document [file mmc1.pdf]

Conflicts of Interest Statement

Manuscript title: Illumina sequencing of the chloroplast genome of common ragweed (*Ambrosia artemisiifolia* L.)

The authors whose names are listed immediately below certify that they have NO affiliations with or involvement in any organization or entity with any financial interest (such as honoraria; educational grants; participation in speakers' bureaus; membership, employment, consultancies, stock ownership, or other equity interest; and expert testimony or patent-licensing arrangements), or non-financial interest (such as personal or professional relationships, affiliations, knowledge or beliefs) in the subject matter or materials discussed in this manuscript.

Authors:

Dr. Eszter Virág

Erzsébet Nagy

Géza Hegedűs

Dr. János Taller

Barbara Kutasy

We declare, that we have no conflict of interest.

Name and signature

Erzsébet Nagy

Dr. Taller János

Barbara Kutasy

Géza Hegedűs

Dr. Eszter Virág

Corresponding author

21/09/2017
